# Impact of chronic kidney disease on the prognosis of transcatheter aortic valve replacement in patients with aortic stenosis: A protocol for systematic review and meta-analysis

**DOI:** 10.1097/MD.0000000000026696

**Published:** 2021-07-23

**Authors:** Jialu Wang, Shidong Liu, Xiangxiang Han, Zunhui Wan, Yang Chen, Hao Chen, Bing Song

**Affiliations:** a The First Clinical Medical College of Lanzhou University, Lanzhou University, Lanzhou, China; b Department of Cardiovascular Surgery, First Hospital of Lanzhou University, Lanzhou, China.

**Keywords:** chronic kidney disease, meta-analysis, prognosis, transcatheter aortic valve replacement

## Abstract

**Background:**

The prognosis of patients with aortic stenosis, in conjunction with chronic kidney disease (CKD), after transcatheter aortic valve replacement (TAVR) remains unclear. This study assessed the impact of CKD, and different stages of CKD, on prognosis of patients undergoing TAVR.

**Methods:**

The protocol was written following the Preferred Reporting Items for Systematic Reviews and Meta-Analyses Protocols statement guidelines. As of June 2021, we performed a comprehensive literature search on studies related to CKD and TAVR, using databases such as PubMed, Embase, Cochrane Library, and Web of Science. Two researchers independently screened the literature, extracted the data, and evaluated the risk of bias in the included studies. Then, Stata 15.0 software was used for meta-analysis.

**Results and Conclusion:**

The purpose of this study was to evaluate the effect of CKD and different stages of CKD on the prognosis of patients with TAVR. It is hoped to provide a comprehensive reference for clinical practice and related clinical trials in the future.

## 1 Introduction

With a rise in the global aging population, aortic stenosis (AS) has become one of the most common valvular diseases.^[1]^ Apart from affecting patient quality of life, severe AS can bring about death, in a relatively short period, if not treated with valve replacement.^[2]^ In the last decade, transcatheter aortic valve replacement (TAVR) has gained popularity as an alternative to surgical aortic valve replacement for patients, who are either inoperable or are at high- to intermediate-risk for surgery.^[3,4]^

Chronic kidney disease (CKD) often coexists with AS, likely due to similar risk factors and pathophysiology.^[5]^ Recently, CKD was reported in approximately 75% of patients with severe AS.^[6]^ Mechanistically, it was demonstrated that CKD accelerates dystrophy calcification in the aortic valve, which contributes to severe AS 10 to 20 years earlier than in the general population.^[7]^ It is well known that CKD is detrimental to the course of valvular heart disease and prognosis of cardiovascular intervention.^[8,9]^ Moreover, the presence of CKD is also shown to increase both short- and long-term mortality in surgical aortic valve replacement patients, with short-term mortality reaching as high as 21%.^[10]^

The prognostic effects of CKD on TAVR, however, remain unclear. Moreover, little is known about the difference of prognosis among different CKD stages. At present, there is no meta-analysis on the outcome of preoperative CKD on long-term prognosis of TAVR. Therefore, our meta-analysis aimed to investigate the outcome of CKD, and different stages of CKD, on the short-, medium-, and long-term prognosis of TAVR patients.

## 2 Materials and methods

### 2.1 Protocol registration

The protocol for this systematic review was registered on INPLASY (INPLASY 202160023) and is available in full on the inplasy.com. This protocol that has been reported is based on the Preferred Reporting Items for Systematic Reviews and Meta-Analyses Protocols (PRISMA-P) guideline.^[11]^

### 2.2 Study selection

Two researchers conducted an extensive and systematic computerized literature review, using the PubMed, Cochrane Library, EMBASE, and Web of Science databases, and searched from the inception of indicated databases till June 2021, using various combinations of the following free text and key terms: “TAVI,” “TAVR,” “transcatheter aortic valve implantation,” “transcatheter aortic valve replacement,” “chronic kidney disease,” “chronic kidney failure,” and “CKD.” To further ensure no relevant publications were overlooked, we also manually searched the list of references for publications that might meet our requirements.

### 2.3 Inclusion and exclusion criteria

1. Population: patients diagnosed with AS and treated with TAVR.
2. Intervention group: patients with AS complicated CKD treated with TAVR.
3. Comparison group: patients with AS without CKD treated with TAVR.
4. Outcome measures: The primary outcome of our study was all-cause mortality after TAVR at the short-(30-day), medium-(1-year), and long-term (2-year) follow-ups. Secondary outcomes included stroke, bleeding, permanent pacemaker implantation, acute kidney injury, and major vascular complications at the short-term (30-day) follow-up. All outcomes after TAVR were defined according to the standard described by Valve Academic Research Consortium.
5. Study design: randomized controlled trial (RCT) and cohort studies.

The exclusion criteria included: repeated publication or overlapping of patients; unclear report or unable to calculate relevant results based on published data; conference, reviews, case reports, and editorials; non-English-language studies.

### 2.4 Data extraction

Two researchers independently screened the literature, extracted the data, and cross-checked it. Differences, if any, shall be resolved through discussion or negotiation with a third party. During literature screening, the title of the literature was first to read. After excluding the irrelevant literature, the abstract and full text were further read to determine whether to include the literature or not. If necessary, contact the original study author via email or telephone to obtain uncertain but important information for the study. The data extraction content mainly includes: study characteristics: first author, country, publication time; patient characteristics: sample size, age, gender, diabetes, hypertension, peripheral vascular disease, chronic pulmonary disease, and left ventricular ejection fraction; surgical characteristics: including surgical approach, valve type, logistic EuroSCORE, and the Society of Thoracic Surgeons score; outcome: including all-cause mortality, cardiovascular mortality, acute kidney injury, bleeding, and major vascular complications; key elements of bias risk assessment.

### 2.5 Quality of evidence assessment

The risk of bias in the included studies was independently evaluated by 2 investigators, and the results were cross-checked. The RCT risk of bias was assessed using the RCT risk of bias assessment tool recommended in the Cochrane Handbook 5.1.0. The Newcastle-Ottawa Scale was used for the bias risk assessment. In case of differences, they shall be resolved through discussion or with the assistance of a third party.

### 2.6 Statistical analysis

The frequency of categorical variables and the standardized means with standard deviations of continuous variables were used for descriptive analysis. The risk ratio and 95% confidence interval of the results were performed using a meta-analysis for random effect models. The evaluation of the heterogeneity of different studies used the Cochrane Q-statistic to calculate the I^2^ values, where < 25%, 25% to 50%, and >50%, respectively, indicated low, medium, and high heterogeneity. Sensitivity analysis further explored significant heterogeneity. Publication bias was assessed by funnel plot asymmetric analysis and Egger regression test. *P* values were bilateral, *P* < .05 was set as statistical significance threshold. Stata15.0 statistical analysis software was used for data analysis.

## 3 Discussion

CKD is a chronic renal structure or function disorder caused by various primary or secondary causes. Its prevalence is increasing year by year, and it has become a global public health problem. Studies have shown that there is a strong association between AS and CKD, and in the inoperable/high-risk patient group of the PARTNER trial, about 70% of patients who received TAVR had moderate (58%) or severe (12%) CKD preoperatively.^[12]^ The frequent association between AS and CKD is the result of a malignant physiopathological process in which renal disease promotes aortic valve calcification and valve stenosis leads to reduced renal blood flow that further impair renal function.^[13]^ Since the first TAVR procedure was successfully performed in 2002, it has become the standard of care for patients with severe AS who cannot undergo surgery and effective alternative treatment for patients with surgically high-risk AS. Currently, with the extension of the TAVR indication to patients with severe AS who are at low surgical risk, the number of operations for TAVR may increase further in the future. Therefore, it is important to identify clinical risk factors independent of prognosis. However, the relationship between the presence of CKD and the clinical prognosis of TAVR patients has always been controversial. Therefore, this study conducted a systematic review of relevant studies, aiming to study the effect of CKD and different stages of CKD on the prognosis of patients with TAVR and to provide an evidence-based basis for clinical practice.

## Author contributions

**Conceptualization:** Zuihui Wan.

**Data curation:** Jialu Wang and Shidong Liu.

**Methodology:** Yang Chen and Hao Chen.

**Project administration:** Bing Song.

**Writing – original draft:** Jialu Wang and Shidong Liu.

**Writing – review &editing:** Zunhui Wan and Xiangxiang Han.
